# Hidden clear cell acanthoma with uncommon dermoscopic findings^☆^^☆☆^

**DOI:** 10.1016/j.abd.2020.06.027

**Published:** 2021-05-15

**Authors:** Marcial Álvarez-Salafranca, Mar García-García, Andrea Montes-Torres, Mariano Ara-Martín

**Affiliations:** Hospital Clínico Universitario Lozano Blesa, Zaragoza, Spain

Dear Editor,

A 73-year-old woman presented with less than a 1-year history of an erythematous, well-delimited and shiny lesion on her left buttock, near the gluteal fold (Fig. 1). Dermoscopic examination showed dotted vessels arranged in a linear pattern involving the entire lesion. These vascular findings are also called metaphorically a ‘string of pearls’ pattern. Another vascular pattern observed on the periphery of the lesion is that of branched vessels with rounded endings. Surprisingly, dermoscopy also revealed multiple rosettes (also known as ‘four-clod dots’) (Fig. 2). Differential diagnoses included mainly clear cell acanthoma, irritated seborrheic keratosis, Bowen´s disease and eccrine poroma. The lesion was completely surgically removed and histopathologic examination revealed an epidermic lesion with marked psoriasiform hyperplasia, in which epidermal cells show clear cytoplasms (Fig. 3). These findings are consistent with the diagnosis of Clear Cell Acanthoma (CCA).

**Figure 1:**
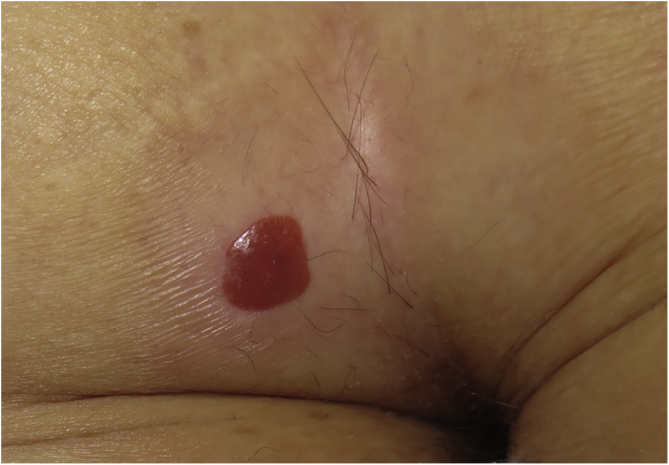
Well delimited erythematous shiny plaque on the left buttock, surrounded by a hypopigmented area.

**Figure 2:**
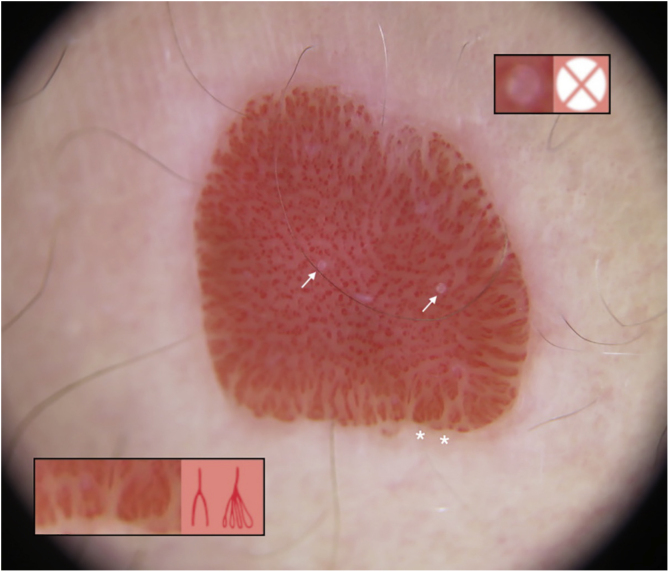
Dermoscopy showed dotted vessels arranged in a linear pattern involving the central part of the lesion (‘String of pearls’ pattern). In addition, branched vessels with rounded endings (white asterisk; bottom inset) and four-clod dots can be observed (white arrows; upper black inset).

**Figure 3:**
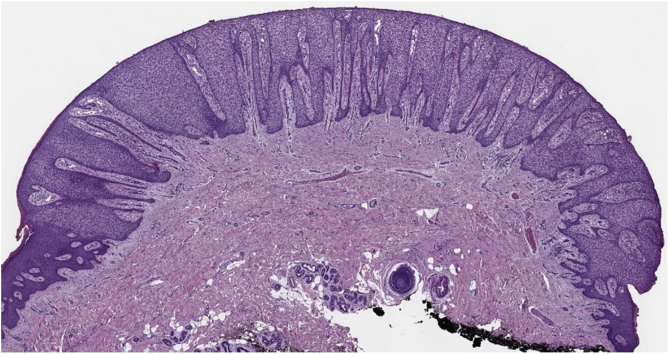
At low power view there is a very well-demarcated lesion with prominent acanthosis and psoriasiform hyperplasia. Clear, pale-staining keratinocytes are characteristically present in this lesion (Hematoxylin & eosin, ×2).

CCA is an uncommon benign epidermal lesion of unknown etiology, although a recent hypothesis suggests its reactive origin. The typical presentation is a red to brown, dome-shaped papule or plaque on the lower extremities, with a peak of age incidence of 60-years.^1^ However, like in this case, it can appear in unusual locations. Clinical differential diagnosis includes a wide range of cutaneous neoplasms, including malignant lesions such as squamous cell carcinoma or amelanotic melanoma. Indeed, it is frequently mistaken clinically for other skin lesions.^1^ In this setting, almost unique dermoscopic findings can be a useful tool to suspect this diagnosis.

On dermoscopy CCA is highly characteristic, showing dotted or glomerular vessels arranged in a linear or serpiginous pattern. These linear arrangements usually adopt a reticular distribution, forming what is known as a ‘string of pearls’ pattern, of which this case is very representative.^2^ In this sense, these stereotypical dermoscopic findings can be helpful in the setting of otherwise nonspecific lesions.^3^ Other dermoscopic findings reported in CCA are, in order of frequency, a pale pink background, a collarette of translucent scales, hemorrhagic areas, orange crusts, and crystalline structures.^2^

Otherwise, branched vessels with rounded terminal endings are not typical of CCA. Moreover, these dermoscopic finding is positively associated with the diagnosis of eccrine poroma, which is one of the main differential diagnoses of CCA.^4^ In the studied patient, they were distributed along the entire periphery of the lesion, showing various morphologies such as a chalice-like or leaf-like shape. This peripheral distribution could suggest that, in this case, vessel type depends more on the viewing angle on dermoscopy than on the morphology of these vessels.

Rosettes are dermoscopic structures only observed under polarized light. They are also called ‘four-clod dots’ and, first believed to be specific for actinic keratosis and squamous cell carcinoma, currently, they are not considered lesion-specific and can also be observed in another type of skin lesions. Rosettes are considered to be an optical effect of crossed polarization by concentric fibrosis or horny material.^5^ To the authors’ knowledge, there are no previous reports showing this structure end CCA.

In conclusion, the authors report a case of CCA with an unusual location, showing uncommon dermoscopic findings, classically associated with other cutaneous lesions such as actinic keratosis or eccrine poroma. Although the ‘string of pearls’ pattern is highly characteristic, CCA can show other dermoscopic features that should be known in order to make an accurate differential diagnosis.

## Financial support

None declared.

## Authors’ contributions

Marcial Álvarez-Salafranca: Approval of the final version of the manuscript; composition of the manuscript; collection, analysis, and interpretation of data; participation in the design of the study; critical review of the literature; critical review of the manuscript.

Mar García-García: Approval of the final version of the manuscript; collection, analysis, and interpretation of data; critical review of the manuscript.

Andrea Montes-Torres: Approval of the final version of the manuscript; collection, analysis, and interpretation of data; critical review of the manuscript.

Mariano Ara-Martín: Approval of the final version of the manuscript; critical review of the manuscript.

## Conflicts of interest

None declared.
